# Impact of discounts on medicine prices

**DOI:** 10.1186/2052-3211-8-S1-O4

**Published:** 2015-10-05

**Authors:** Peter Schneider, Sabine Vogler

**Affiliations:** 1 WHO Collaborating Centre for Pharmaceutical Pricing and Reimbursement Policies, Health Economics Department, Gesundheit Österreich GmbH (Austrian Public Health Institute), Vienna, 1010, Austria

## Problem statement

Several European countries implemented external price referencing (EPR) as a pricing policy for medicines, and as such they use price data from other countries as a benchmark to determine their medicine prices. In current EPR practice undiscounted official list prices are taken as a reference. There is some debate as to which extent discounts have an impact on medicine prices. The aim of the study was to investigate whether and how much medicine prices would change if discounts were considered.

## Methodology

Ex-factory prices of 30 medicines (15 medicines from the out-patient sector and 15 medicines from the in-patient sector) that accounted for high expenditure for public payers in Austria were surveyed for 16 European countries (Belgium, Denmark, Finland, France, Germany, Greece, Hungary, Ireland, Italy, the Netherlands, Portugal, Sweden, Slovakia, Spain and the UK). In addition to these official list prices, the statutorily discounted ex-factory medicines in Germany were collected and compared.

## Results

Overall, for the 30 selected medicines, Germany was the country that had most frequently the highest prices (in 40% of the 30 medicines), followed by Sweden (23%) and Denmark (13%). If discounted medicines instead of list prices were considered for Germany, Sweden was most frequently the highest-priced country (37% of the medicines), followed by Denmark (17%). Together with Austria and the UK, Germany ranked third (10%). In a comparison of list prices only, Swedish prices were in the fourth quartile in 85% and German prices in 80% of the medicines. Considering discounted prices for Germany, their prices ranked in the fourth quartile in only 30% of all medicines, compared with 89% for Sweden and 47% for Austria and Denmark respectively. Among the analysed medicines the impact on the price level was strongest for medicines with the comparably highest prices.

## Conclusions

In several European countries pharmaceutical manufacturers grant confidential rebates to public payers [1], leading to lower actual prices compared with list prices. Since EPR is usually based on official list prices, countries risk over-paying. This case study in which statutory, published discounts for a sole country were considered suggests a high impact on medicine prices. Taking discounts into account, medicine price levels in Germany were comparably lower. A consideration of discounts in further countries is likely to show further reductions in medicine price levels. Disclosure of discounts might help improve pricing policies in European countries.

## References

[B1] Vogler S Zimmermann N Habl C Piessnegger J Bucsics A Discounts and rebates granted to public payers for medicines in European countries Southern Med Review 2012 5 1 38 46 23093898 PMC3471187

